# SelfCheck-Eval: A multi-module framework for zero-resource hallucination detection in large language models

**DOI:** 10.1016/j.patter.2026.101569

**Published:** 2026-06-05

**Authors:** Diyana Muhammed, Giusy Giulia Tuccari, Gollam Rabby, Sören Auer, Sahar Vahdati

**Affiliations:** 1 TIB—Leibniz Information Centre for Science and Technology, Hannover, Germany; 2 L3S Research Center, Leibniz University Hannover, Hannover, Germany; 3 Department of Mathematics and Computer Science, University of Catania, Catania, Italy; 4 National Research Council, Institute of Cognitive Science and Technology, Catania, Italy

**Keywords:** hallucination detection, large language models, consistency and uncertainty quantification, context-based verification

## Abstract

Large language models (LLMs) have achieved considerable progress across diverse applications, yet their tendency to generate incorrect or fabricated content, commonly termed hallucinations, remains a fundamental obstacle to reliable deployment in high-stakes domains. Existing detection benchmarks are confined to general-knowledge settings, leaving specialized fields, where accuracy is important, underexplored. To address this gap, we introduce the American Invitational Mathematics Examination (AIME) Math Hallucination dataset, a benchmark for evaluating mathematical reasoning hallucinations, and propose SelfCheck-Eval, an LLM-agnostic, black-box detection framework compatible with open- and closed-source LLMs. The framework integrates three independent modules, semantic, specialized detection, and contextual consistency, into a suitable architecture. Systematic evaluation reveals a noticeable performance gap: existing methods perform well on biographical content but struggle with mathematical reasoning, a deficit that continues across natural language inference (NLI) fine-tuning, preference learning, and process supervision paradigms. These findings expose fundamental limitations of current approaches and motivate the development of specialized, black-box-compatible methods for trustworthy LLM deployment.

## Highlights

• SelfCheck-Eval detects hallucinations without accessing LLM internals
• AIME Math benchmark introduced for mathematical hallucination detection
• All current methods fail to validate correct mathematical reasoning reliably
• Hallucination detection in mathematics requires more than statistical learning

## The bigger picture

Artificial intelligence systems based on large language models are increasingly deployed in high-stakes settings such as healthcare, legal analysis, and scientific research. Yet these systems carry a fundamental flaw: they can generate text that sounds definitive while being factually wrong, known as hallucination. Detecting these errors is therefore as critical as building the systems themselves. Most existing detection tools have been tested on general, everyday knowledge, leaving domains that require precise logical reasoning, such as mathematics, largely unexplored. In this work, we show that current hallucination detection methods break down when applied to mathematical reasoning, struggling not only to catch errors but to identify correct solutions. This failure continues even after fine-tuning on specialized mathematical data, pointing to a deeper structural limitation. To support this research, we present a framework and benchmark that provide a realistic testbed for evaluating AI reliability in formal reasoning contexts.

## Teaser

Large language models are powerful but tend to generate convincing yet incorrect content, a problem called hallucination. While tools exist to catch these errors in general knowledge, they fail in mathematical reasoning, unable to reliably distinguish correct solutions from inferior ones. Muhammed, Tuccari, Rabby, et al. introduce a new mathematical benchmark and a detection framework, revealing that this failure persists across all tested approaches, signaling a fundamental gap that demands purpose-built solutions for AI reliability in technical domains.

## Introduction

Large language models (LLMs)^1^ have revolutionized natural language processing (NLP), excelling in tasks like summarization, question answering, and dialogue generation. However, despite their impressive capabilities, LLMs can generate outputs that appear plausible but are factually incorrect, a phenomenon referred to as hallucination.^2^ Among the various types of hallucinations, input conflicting, context conflicting, and fact conflicting, the latter poses the most significant challenges due to its potential to spread misinformation across critical domains such as healthcare, finance, or education. Detecting and mitigating hallucinations is crucial to ensuring the reliability of LLMs in these sensitive areas.^3^

Hallucination detection methods must be evaluated across different domains to understand their generalization capabilities. We focus on two complementary domains: biographical and mathematical reasoning. In biographical contexts, hallucinations appear as incorrect facts, while in mathematical reasoning, hallucinations involve flawed logic in multi-step problem solving. This tests whether detection methods work across content types or domain-specific approaches, a critical question for reliable LLM deployment.

Traditional hallucination detection approaches^4^ focus primarily on static claim-evidence pairs and cannot handle the dynamic context intrinsic to LLM-generated content. Moreover, existing benchmarks for hallucination detection^5^ primarily assess basic factual consistency in general domains, often neglecting the unique challenges posed by complex mathematical reasoning where both logical consistency and factual correctness are required. This narrow scope leaves critical gaps in understanding how different detection paradigms perform across domains with varying reasoning requirements, particularly in mathematical contexts where current methods may face fundamental limitations. As shown in Table 1, existing datasets focus primarily on general domains and lack sampled LLM responses, which is a major factor for our evaluation approach.

**Table 1:** Comparison with existing hallucination detection datasets

Dataset	Source	Domain	Annotation	Logical reasoning	LLM sampled	Usefulness
TruthfulQA^6^	general	text	manual	✗	✗	✗
HaluEval^7^	text	general	manual	✗	✗	✗
HADES^8^	token level	general	crowdsourced	✗	✗	✗
FACTCHD^9^	KGs and text	general	manual	✗	✗	✗
WikiBio^10^	text	biography	manual	✗	✓	✓
AIME Math(ours)	math problems	mathematics	AI-human	✓	✓	✓

Our AIME Math includes complex mathematical domains, such as number theory, geometry, algebra, and others. KGs, knowledge graphs.

To address these gaps, we introduce SelfCheck-Eval, a systematic comparative framework that evaluates three distinct detection modules across biographical and mathematical domains.

Our methods are designed for zero-resource black-box hallucination detection. The black-box constraint means we operate without access to LLM internals. The zero-resource constraint means we do not use external knowledge bases or fact-checking systems, following the definition introduced by Potsawee et al.^2^ Instead, our methods rely solely on analyzing multiple text outputs generated by the LLM being evaluated. This design makes them applicable to any LLM, including closed-source systems.

**Figure 1:**
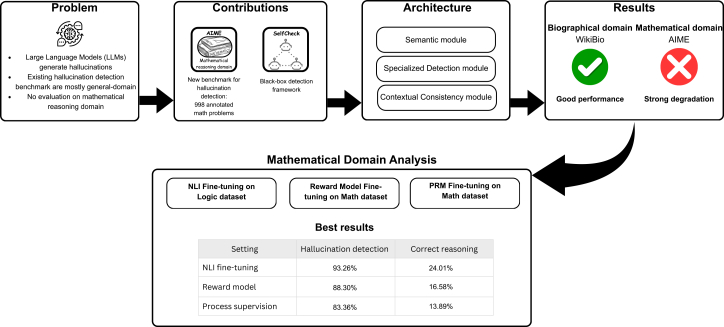
Overview of the hallucination detection experiment conducted within the SelfCheck-Eval framework

Our framework examines (1) statistical approaches based on frequency analysis and response consistency, (2) neural methods using fine-tuned LLMs for entailment detection, and (3) contextual approaches reducing LLM reasoning capabilities through structured prompting. Rather than proposing a unified detection system, we provide empirical insights into how different methods perform when applied to domains with varying complexity and reasoning requirements. In Figure 1, each detection approach independently evaluates LLM responses and provides hallucination scores.

Our investigation proceeds in two phases. First, we systematically evaluate three detection paradigms across biographical (WikiBio) and mathematical (American Invitational Mathematics Examination [AIME]) domains. Second, given the consistent failures observed in mathematical reasoning validation, we investigate whether specialized fine-tuning on mathematical datasets can overcome these limitations through natural language inference (NLI) fine-tuning, reward modeling, and process supervision approaches. To enable this systematic evaluation, we introduce the AIME Math Hallucination benchmark. Unlike previous approaches that rely on artificially injected hallucinations, our benchmark uses mathematical reasoning errors naturally generated by state-of-the-art LLMs, validated through human-AI collaboration. This provides a realistic testbed for assessing detection methods on authentic mathematical reasoning challenges. Our evaluation across the WikiBio and AIME Math datasets reveals significant performance variations between domains. While detection methods achieve strong performance on biographical content, they exhibit consistently asymmetric behavior in mathematical reasoning. These methods effectively detect incorrect solutions but systematically struggle to validate correct reasoning. This pattern emerges across different approaches, suggesting fundamental challenges in current methods when applied to mathematical domains. The main contributions of this work are as follows.

• We provide a systematic comparative analysis of zero-resource black-box hallucination detection paradigms across domains, revealing significant performance variations between biographical and mathematical content.
• We introduce the AIME Math Hallucination benchmark, which features naturally occurring mathematical reasoning errors generated by state-of-the-art LLMs, providing a realistic evaluation testbed beyond existing datasets that rely on artificial hallucination injection.
• We observe consistent asymmetric performance patterns across multiple detection paradigms in mathematical reasoning, with high sensitivity to errors but systematic difficulty validating correct solutions.
• We evaluate specialized fine-tuning approaches, including NLI-based fine-tuning, reward modeling, and process supervision, finding that the mathematical reasoning challenge continues across training paradigms.

## Related work

LLMs demonstrate remarkable abilities in understanding and executing user instructions.^11^ However, they frequently generate misleading outputs, termed hallucinations.^12^ These hallucinations are categorized into input-conflicting, context-conflicting, and fact-conflicting types, with fact-conflicting hallucinations posing the most significant challenges due to their potential to propagate misinformation.^9^

Previous research has investigated hallucinations across various NLP tasks, including summarization^13^ and dialogue generation,^14^ as well as other applications.^15^ Self-consistency decoding has shown promise in improving reasoning accuracy by leveraging multiple reasoning paths,^16^ yet its effectiveness in detecting factual inaccuracies remains underexplored. Despite these advances, current methodologies exhibit critical limitations: a lack of standardized evaluation metrics for fact-conflicting hallucinations, an absence of domain-specific benchmarks for mathematical reasoning, and insufficient focus on black-box detection methods suitable for API-accessible LLMs. Frequency-based methods rely on statistical patterns and token probability estimation from multiple LLM outputs, such as *n*-gram analysis and distributional consistency measures. Neural approaches leverage fine-tuned LLMs for NLI or entailment detection, often requiring supervised fine-tuning on domain-specific data. Contextual methods exploit the reasoning capabilities of LLMs themselves through carefully designed prompts and consistency evaluation. While each paradigm has demonstrated effectiveness in specific scenarios, comprehensive comparative analysis across different domains, particularly in mathematical reasoning, remains underexplored. Perturbation-based approaches^17^ and synthetic data generation methods^8^ artificially modify factual texts to create hallucinatory data, potentially missing the natural variation patterns of real LLM hallucinations. White-box methods^9^ require supervised data and access to internal LLM states, whereas self-evaluation approaches^18^ show limited generalizability across different tasks and domains.

Mathematical reasoning presents unique challenges for hallucination detection that are not adequately addressed by existing approaches designed for general-domain text. Mathematical content requires both logical consistency across multi-step derivations and factual correctness of individual claims, making it qualitatively different from biographical or narrative text, where surface-level inconsistencies may suffice for detection. Existing works in mathematical reasoning typically employ standard math benchmarks such as GSM8K^19^ or MATH^20^ and create hallucinated data through artificial injection methods rather than analyzing naturally occurring mathematical reasoning errors.

Recent work introduces the fine-grained process reward model (FG-PRM),^21^ which proposes a fine-grained taxonomy of six hallucination types for mathematical reasoning tasks. Their approach focuses on step-level detection using specialized PRMs^22^ trained on synthetically generated hallucination data and validated on human-annotated solutions. However, their synthetic data generation involves systematically injecting predefined hallucination types into correct solutions, which may not capture the authentic distribution of naturally occurring mathematical reasoning errors that emerge during actual LLM deployment.

Farquhar et al.^23^ introduce semantic entropy as a statistical framework for hallucination detection. The method estimates uncertainty by sampling multiple responses, clustering them according to their semantic similarity via bidirectional entailment, and computing entropy over the cluster distribution. While this approach performs well for open-domain question answering, its evaluation on mathematical reasoning was limited to SVAMP,^24^ a dataset of elementary grade school word problems. The application of more complex mathematical reasoning presents unique challenges. In mathematical contexts, solutions often involve long reasoning chains followed by numerical answers. Two solutions may appear semantically close because they share intermediate reasoning yet still diverge in the final step, leading to different outcomes. Conversely, reasoning chains with different surface forms can nonetheless converge to the same correct answer. Thus, when applying semantic entropy to mathematical reasoning, both the coherence of the reasoning process and the correctness of the final answer need to be considered.

The effectiveness of different detection paradigms across mathematical-domain versus general-domain content remains underexplored, with most evaluations focusing on single-domain performance rather than systematic cross-domain analysis. Our work addresses this gap by introducing a systematic comparative analysis framework that evaluates three distinct detection paradigms across both biographical and mathematical domains. Rather than proposing a unified detection method, we provide empirical insights into the relative strengths and limitations of different approaches when applied to domains with varying complexity and reasoning requirements.

## Datasets and metrics

In this section, we present the datasets employed in our comprehensive analysis for detecting hallucinations across different domains. Our evaluation framework encompasses two complementary benchmarks that together provide a robust assessment of hallucination detection capabilities.

We begin by examining WikiBio,^2^ an established general-knowledge domain benchmark for hallucination detection, which serves as our baseline for comparative analysis. Subsequently, we introduce AIME Math, a benchmark specifically designed for evaluating and detecting hallucinations in mathematical reasoning tasks. This new benchmark addresses the critical gap in domain-specific evaluation tools for mathematical domains. Finally, we provide a detailed description of the AIME Math construction methodology, highlighting the design principles and validation procedures that ensure its effectiveness in mathematical hallucination detection. The sample distributions of these datasets are summarized in Table 2.

**Table 2:** Sample distribution of the experimental datasets

Dataset	Major inaccurate	Minor inaccurate	Accurate	Total
WikiBio	761	516	631	1,908
AIME Math	823	38	137	998

### WikiBio dataset

The WikiBio dataset represents a well-established benchmark for hallucination detection in general-knowledge domains, comprising 238 Wikipedia paragraphs paired with corresponding tabular data. The dataset construction relied on synthetic passages generated with GPT-3.5^2^ through a standardized prompt template: “This is a Wikipedia passage about [concept]:.”

This approach ensures consistent generation conditions while covering diverse biographical topics. The resulting dataset contains 1,908 sentences with an average length of 184.7 tokens per sentence. Each sentence underwent comprehensive factuality annotation using a three-level classification system: major inaccurate (39.9%), minor inaccurate (33.1%), and accurate (27.0%). The annotation process demonstrates robust inter-annotator reliability, with Cohen’s *κ* scores of 0.595 for the full three-class distinction and 0.748 for binary accurate/inaccurate classification. For quality assurance, 201 sentences received dual annotation, with disagreements resolved using a conservative worst-case labeling approach. The dataset’s design enables both sentence-level and passage-level evaluation. Passage-level hallucination scores, computed by averaging constituent sentence scores, range up to +1.0, indicating complete factual inaccuracy. This multi-granular annotation framework provides a comprehensive foundation for evaluating hallucination detection methods across different levels of textual analysis.

### AIME Math Hallucination benchmark

The AIME Math Hallucination dataset addresses the critical need for domain-specific benchmarks in mathematical reasoning by leveraging problems obtained from the AIME. Our construction methodology follows a systematic three-phase approach designed to ensure both scalability and annotation quality.

#### Data collection and response generation

The initial phase involved a comprehensive collection of human solutions for all 998 AIME problems, establishing verified ground-truth solutions with detailed explanations for subsequent evaluation. To capture the variability inherent in LLM mathematical reasoning, we generated five distinct responses per problem using GPT-4o with zero-shot (ZS) prompting. This multi-response approach enables robust evaluation of consistency patterns and provides sufficient sample diversity for statistical analysis of hallucination detection methods.

#### Human-AI collaboration annotation

Our annotation methodology combines automated processing with human oversight to achieve both efficiency and accuracy. For each problem, we randomly selected one response from the five generated samples and evaluated its correctness against the human-provided ground-truth solution. The initial categorization employed a binary matching criterion: responses producing answers consistent with the ground truth were preliminarily classified as potentially accurate, while those with incorrect final answers were automatically designated as major inaccurate.

To refine this initial classification, we implemented a hybrid human-AI collaboration framework that uses GPT-3.5 for systematic answer matching and is supplemented by human annotator review for ambiguous cases. This approach leverages the efficiency of automated processing while maintaining the nuanced judgment necessary for mathematical content evaluation. The final quality filtering required human annotators to verify all responses and assign them to one of three categories. Responses were labeled as accurate when both the final answer and the reasoning steps matched the human solution. Responses received a minor inaccurate label when the final answer was correct but the reasoning contained small calculation errors or logical missteps. Finally, responses were marked as major inaccurate when they contained completely incorrect reasoning or produced an incorrect final answer. Out of 998 responses, 823 were major inaccurate, 137 were accurate, and 38 were minor inaccurate. These annotations form the foundation for assessing the LLM’s mathematical reasoning and hallucination detection capabilities.

### Evaluation metrics

We evaluate hallucination detection using two complementary metrics designed to capture different aspects of model performance under class-imbalance conditions.

#### AUC-PR

We compute the area under the precision-recall curve (AUC-PR) separately for nonfactual and factual detection. AUC-PR is more informative than ROC-AUC (receiver operating characteristic area under the curve) under severe class imbalance, as it focuses on the performance of the minority class rather than being dominated by the majority class. Given the substantial imbalance in our AIME Math dataset (6:1 ratio of inaccurate to accurate responses), we interpret AUC-PR scores through relative comparisons across methods rather than absolute thresholds. High nonfactual AUC-PR indicates reliable hallucination detection, while high factual AUC-PR reflects the ability to recognize correct outputs.

#### Ranking

We report the Pearson correlation coefficient (PCC) between model confidence scores and gold-standard factuality labels. This metric evaluates how well models rank outputs by factuality, independent of specific classification thresholds. A strong positive correlation indicates that models consistently assign higher confidence to factual content and lower confidence to hallucinated content, which is crucial for deployment scenarios where relative confidence matters more than binary classification.

Together, these metrics provide complementary perspectives: AUC-PR captures threshold-dependent classification performance for each class, while PCC evaluates threshold-independent ranking quality. This dual evaluation ensures a comprehensive assessment of both detection sensitivity and confidence calibration across our imbalanced datasets.

## Methods

Detailed descriptions of the experimental procedures, including mathematical formulations of the three modules and specific hyperparameters for the fine-tuned models, are provided in the supplemental information.

**Figure 2:**
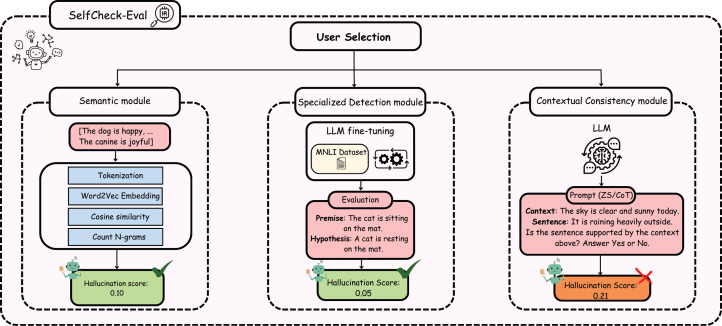
Overview of the SelfCheck-Eval pipeline with the approaches for hallucination detection: Semantic module, specialized detection module (pre-trained and fine-tuned), and contextual consistency module

Existing hallucination detection methods face a fundamental trade-off: approaches optimized for catching obvious errors often fail to validate subtle correctness, while methods excelling at recognizing accurate content frequently miss sophisticated hallucinations. To address this challenge, we introduce SelfCheck-Eval, a framework that evaluates three independent detection strategies, each targeting different aspects of this problem. An overview of the full SelfCheck-Eval framework and the three detection methods is provided in Figure 2. The sampled responses were generated by querying the target LLM multiple times with the same query. One response serves as the target to evaluate; the others serve as sampled references. Each method produces a hallucination score in [0, 1].

The semantic module detects hallucinations by identifying content that deviates from expected distributional patterns, combining frequency-based analysis with semantic similarity. The specialized detection module frames the problem as NLI, leveraging fine-tuned LLMs to assess the logical consistency between responses and evidence. The contextual consistency module exploits the reasoning capabilities of LLMs to evaluate whether generated content aligns with sampled contextual information.

Each module operates independently, providing complementary perspectives on the same detection task. This allows us to evaluate which approaches are most effective across different domains and types of hallucinations, as illustrated in our experimental pipeline (Figure 2). The following subsections detail the technical implementation of each module (additional details regarding dataset structures, fine-tuning configurations, and complete prompt templates are available in Notes S1–S3).

### Semantic module

The semantic module operates on the assumption that hallucinated content exhibits unusual distributional patterns compared to typical responses for similar queries. While conceptually simpler than contextual embedding approaches, this frequency-based model offers computational efficiency and explicit interpretability.

Formally, given sampled generations $\left\{S_{1},\ldots ,S_{N}\right\}$ and the target response *R*, we extract all tokens and build a vocabulary *V*. The target response *R* is included in the sampled data as a smoothing mechanism, incrementing each of its token counts by one so that *R* is contextualized within the broader distribution. For each token t∈V, let count(t) denote its frequency in the combined samples, and let

$$\text{token}\_\text{count}=\sum_{t\in V}\text{count}\left(t\right)$$

be the total number of observed tokens. We estimate smoothed unigram probabilities using Laplace smoothing with parameter k>0 and set k=1 in all experiments. The resulting smoothed probability is

(Equation 1) $$\hat{p}\left(t\right)=\frac{\text{count}\left(t\right)+k}{\text{token}\_\text{count}+k\;\left|V\right|}\text{.}$$

To capture semantic variation, we extend this model using Word2Vec embeddings.^25^ For tokens *t* and $t^{\prime }$ with embedding vectors $v_{t}$ and $v_{t^{\prime }}$, we compute their cosine similarity and define the semantic similarity

$$\text{similar}\left(t\right)=\left\{t^{\prime }\in V:\text{similarity}\left(t,t^{\prime }\right)\geq \theta \right\}\text{,}$$

where θ=0.9 ensures that only highly similar tokens contribute to the aggregated probability. The semantic probability of token *t* is then

(Equation 2) $$\tilde{p}\left(t\right)=\sum_{t^{\prime }\in \text{similar}\left(t\right)}\hat{p}\left(t^{\prime }\right)\text{.}$$

The negative log likelihood of token *t* is defined as

$$\text{NLL}\left(t\right)=-\log\tilde{p}\left(t\right)\text{,}$$

with higher values indicating tokens that are improbable under the sampled distribution and therefore potential hallucinations.

To quantify hallucination risk at the sentence level, consider a sentence with tokens $\left\{t_{1},\ldots ,t_{n}\right\}$. We compute

(Equation 3) $$\text{AvgNLL}=-\frac{1}{n}\sum_{i=1}^{n}\log\left(\tilde{p}\left(t_{i}\right)\right),\text{MaxNLL}=-\min_{i}\log\left(\tilde{p}\left(t_{i}\right)\right)\text{,}$$

where *n* is the number of tokens in the sentence, AvgNLL captures overall plausibility by averaging across all tokens, and MaxNLL isolates the least probable token, highlighting localized irregularities.

At the document level, sentence-level scores are aggregated as

(Equation 4) $$\text{DocAvgNLL}=\frac{1}{m}\sum_{j=1}^{m}{\text{AvgNLL}}^{\left(j\right)},\text{DocMaxAvgNLL}=\frac{1}{m}\sum_{j=1}^{m}{\text{MaxNLL}}^{\left(j\right)}\text{,}$$

where *m* is the number of sentences. While the two metrics provide complementary perspectives, our evaluation adopts MaxNLL as the primary criterion, owing to its superior effectiveness in distinguishing hallucinated from factual content.

### Specialized detection module

The specialized detection module frames hallucination detection as an NLI problem: if a generated sentence contradicts or is not entailed by relevant context, it likely contains hallucinations. This formulation enables transfer learning from existing NLI datasets and leverages the logical reasoning capabilities developed in these tasks. Technical details for the specific datasets used (LogiQA, RewardMATH, Math-Shepherd, and PRM800K) are provided in Note S3.

We fine-tune LLMs on the MultiNLI^26^ dataset using QLoRA^27^ for efficient adaptation. The fine-tuning objective minimizes cross-entropy loss for the three-way classification task: entailment, neutral, and contradiction.

For hallucination detection, we assess each generated sentence against multiple context passages. The context passages $P_{j}$ correspond to alternative LLM generations for the same query. The fine-tuned LLM predicts the NLI relationship between the sentence and each context passage. We then map these predictions to factuality categories: entailment indicates accurate content, neutral suggests minor inaccuracies, and contradiction signals major inaccuracies. The final factuality score for sentence $s_{i}$ is computed as

$$S_{i}=\frac{1}{\left|P\right|}\sum_{j=1}^{\left|P\right|}f_{\text{score}}\left(s_{i},P_{j}\right)\text{,}$$

where $f_{\text{score}}$ maps NLI predictions to numerical values and |P| is the number of context passages.

This approach tests whether logical consistency learned from general NLI tasks transfers effectively to domain-specific hallucination detection, particularly in mathematical reasoning, where logical coherence is paramount.

### Contextual consistency module

The contextual consistency module leverages the reasoning capabilities of LLMs to directly assess whether generated content is supported by available context. Unlike the previous methods that rely on statistical patterns or learned NLI mappings, this approach exploits the natural language understanding and reasoning abilities of LLMs themselves.

Following Potsawee et al.,^2^ each sentence $s_{i}$ in the primary LLM output is evaluated against the set of alternative passages $\left\{P_{1},\ldots ,P_{\left|P\right|}\right\}$ associated with the same query. These passages also correspond to stochastically generated responses from the same LLM prompted with the same query. Given a sentence $s_{i}$ and a sampled passage $P_{j}$, the LLM is queried with the following template:

Context: $P_{j}$
Sentence: $s_{i}$
Question: Is the sentence supported by the context above?
Answer yes or no.

We consider two prompting strategies. In the ZS setting, the LLM receives only the structured input above (the complete prompt templates for both ZS and chain-of-thought [CoT] strategies are documented in Note S1). In the CoT setting, the prompt is extended with instructions encouraging the LLM to provide intermediate reasoning before giving a final binary answer. LLM outputs are post-processed into numerical scores: yes ⟼0.0 (accurate), no ⟼1.0 (inaccurate), and non-conforming responses ⟼0.5 (ambiguous). The final factuality score for sentence $s_{i}$ is computed as

$$S_{i}=\frac{1}{\left|P\right|}\sum_{j=1}^{\left|P\right|}f_{\text{score}}\left(s_{i},P_{j}\right)\text{,}$$

where $f_{\text{score}}$ maps the LLM’s judgment into {0.0,0.5,1.0} and |P| denotes the number of passages. For the AIME Math dataset, we evaluate complete solutions rather than sentence-level ones due to the difficulty of segmenting mathematical text.

## Experiments

### Setup

#### LLMs

We evaluate our approach using a diverse set of LLMs, covering both proprietary and open-source systems. The selected LLMs include GPT-4,^28^ GPT-3.5,^29^ Phi-3-mini,^30^ Llama 3.1 (8B),^31^ Mistral (7B),^32^ and Gemma (7B).^33^ For benchmarking, we also include RoBERTa-large,^34^ which provides a strong reference point on natural language understanding tasks. This selection enables evaluation of hallucination detection across a broad spectrum of LLM families and capacities.

#### Implementation details

For GPT-4 and GPT-3.5, we rely on OpenAI’s API (December 2024). Responses are generated with a temperature of 0.6, balancing diversity and consistency, and a maximum token limit of 2,048 to ensure efficiency. All experiments are conducted on a computational setup comprising one NVIDIA A500 GPU and two NVIDIA 2080Ti GPUs. For comparability, temperature and decoding settings are standardized across LLMs.

#### NLI fine-tuning

We fine-tuned LLaMA 3.1 8B,^31^ Mistral 7B,^32^ Gemma 7B,^33^ and Phi-3-mini^30^ on the MultiNLI dataset.^26^ All LLMs were fine-tuned with QLoRA 4-bit quantization and LoRA adapters (r=16, α=32, dropout = 0.05). Fine-tuning was performed for three epochs with a learning rate of 2 × 10^−4^, a batch size of 8, and AdamW as the optimizer.

### Evaluation methods

#### Semantic module

The semantic module estimates lexical similarity between generated responses and sampled passages using semantic *n*-gram overlap. It provides a lightweight, frequency-based baseline for hallucination detection.

#### Specialized detection module

The specialized detection module is implemented in two variants: (1) an NLI-based prompting approach, where pre-trained LLMs are queried without fine-tuning and (2) fine-tuned LLMs on the MultiNLI dataset to improve sentence-level factuality judgments. Both variants enable entailment-contradiction reasoning for hallucination detection.

#### Contextual consistency module

The contextual consistency module evaluates whether a sentence is supported by retrieved context passages. We test two prompting strategies: ZS, where the LLMs directly answer based on structured input, and CoT, which encourages intermediate reasoning before the final answer.

#### SelfCheckGPT baseline

For comparison, we include SelfCheckGPT,^2^ a black-box hallucination detection method that samples multiple generations from an LLM and evaluates factual alignment across them. This established approach serves as our primary comparison baseline for validating the effectiveness of our proposed modules. Specifically, we compare against Unigram, BERTScore, SelfCheckGPT-Fine-tuned, and SelfCheckGPT-Prompt.

## Results

We evaluate the effectiveness of SelfCheck-Eval on the WikiBio dataset and on AIME Math, a benchmark proposed in this work. Table 3 reports the overall performance across the three modules: the semantic module, the specialized detection module, and the contextual consistency module. The following subsections provide a detailed analysis of their individual contributions.

**Table 3:** Consolidated comparison of methods, LLM sizes, and prompting strategies on the WikiBio and AIME Math datasets

Method	LLM	Size	Prompt	WikiBio dataset			AIME Math dataset		
				Nonfactual	Factual	Ranking	Nonfactual	Factual	Ranking
SelfCheckGPT (unigram)	–	–	–	85.63	58.47	64.71	85.69	14.91	–
SelfCheckGPT (BERTScore)	–	–	–	85.63	58.47	64.71	**88.51**	**17.82**	–
Semantic module (Word2Vec)	–	–	–	**86.97**	**59.02**	**65.88**	87.24	14.62	11.94
Semantic module (BERT)	–	–	–	**87.02**	56.20	64.69	87.03	14.32	10.54
SelfCheckGPT (fine-tuned)	DeBERTa-v3	–	–	92.50	66.08	74.14	87.64	17.45	5.32
Specialized detection module (pre-trained)	RoBERTa-large	340 M	ZS	76.44	31.20	6.07	–	–	–
	Phi-3	3.8 B	ZS	**89.27**	**56.26**	**61.88**	90.34	19.16	12.75
	Llama 3.1	8 B	ZS	78.90	37.12	34.25	87.76	21.10	3.16
	Mistral	7 B	ZS	86.13	58.60	41.15	91.33	17.11	2.57
	Gemma	7 B	ZS	75.41	30.87	4.80	**93.38**	**20.38**	**21.76**
	Phi-3	3.8 B	CoT	75.08	29.67	13.79	87.76∗	21.10∗	3.16∗
	Llama 3.1	8 B	CoT	63.88	21.75	–	85.33	13.13	–
	Mistral	7 B	CoT	81.91∗	46.10∗	43.49∗	91.33	17.11	2.57
	Gemma	7 B	CoT	70.97	25.99	–	90.07	13.00	0.50
	RoBERTa-large	340 M	CoT	73.86	31.47	13.02	–	–	–
Specialized detection module (fine-tuned)	Phi-3	3.8 B	–	92.87	65.25	73.54∗	**93.38**	**20.38**	**21.76**
	Llama 3.1	8 B	–	76.85	29.71	8.91	79.63	9.74	–
	Mistral	7 B	–	**92.68**	**67.10**	**75.63**	92.91∗	17.37∗	20.05∗
	Gemma	7 B	–	83.47	43.12	50.98	82.58	10.70	–
SelfCheckGPT (prompt-based)	Mistral	7 B	ZS	91.31	62.76	74.46	92.15	45.31	17.19
	gpt3.5	–	ZS	93.42	67.09	78.32	**95.66**	56.95∗	30.08∗
Contextual consistency module (prompt-based)	Llama 3.1	8 B	ZS	92.85	70.73	76.54	94.79	37.75	29.67
	gpt4o	–	ZS	**94.00**	**74.11**	77.48∗	93.93	24.26	21.13
	Mistral	7 B	CoT	91.74	64.01	75.40	93.63	37.98	22.51
	Llama 3.1	8 B	CoT	93.64∗	70.26∗	**78.48**	91.77	24.89	14.23
	gpt3.5	–	CoT	90.59	62.11	72.17	95.28∗	**57.62**	25.42
	gpt4o	–	CoT	94.14	74.95	76.33	94.89	30.58	**30.68**

Reported metrics include nonfactual (AUC-PR), factual (AUC-PR), and ranking (PCC). The best result is shown in bold and the second best result is denoted with an asterisk (detailed results for all model sizes are available in Tables S4 and S5 in Note S5). M, million; B, billion.

### Semantic module

The semantic module, which combines frequency-based unigram probabilities with Word2Vec semantic similarity, demonstrates domain-dependent effectiveness with markedly different performance characteristics across the two benchmarks.

On the WikiBio dataset, the semantic module achieves consistent but incremental improvements over frequency-based baselines. For nonfactual detection, it reaches an AUC-PR of 86.97, a 1.34-point improvement over the SelfCheckGPT unigram baseline (85.63). The gains are more modest on factual detection, with 59.02 compared to 58.47 for both unigrams. Ranking correlation shows a similar pattern, reaching 65.88 versus 64.71 for the baselines, representing a 1.17-point increase. The results suggest that Word2Vec semantic enhancement provides some benefit over pure frequency-based approaches for biographical text, though the gains are limited relative to the computational overhead of semantic similarity calculations.

The results change dramatically when transitioning to the AIME Math dataset. While nonfactual detection remains relatively stable at 87.24 (compared to 85.69 for the unigram baseline), both factual detection and ranking performance collapse severely. Factual detection drops to just 14.62, compared to 59.02 on WikiBio, representing one of the most severe performance drops observed across domains, while ranking correlation falls to 11.94. We additionally evaluated a variant of this module using BERT-based contextual embeddings (all-MiniLM-L6-v2) in place of Word2Vec for token-level semantic similarity. This BERT variant achieves comparable nonfactual detection (87.03 on AIME Math, 87.02 on WikiBio) but does not address the factual detection limitations (14.32 on AIME Math, 56.20 on WikiBio). Given similar performance and Word2Vec’s lower computational requirements, we retain Word2Vec as our primary implementation. Complete results are reported in Table 3.

In contrast, SelfCheckGPT BERTScore, using sentence-level contextual embeddings, achieves superior performance on AIME Math with 88.51 nonfactual and 17.82 factual detection, indicating that sentence-level contextual approaches outperform token-level frequency methods in mathematical domains.

This substantial performance degradation highlights a fundamental limitation of frequency-based approaches in mathematical domains. While the semantic module can effectively identify distributional anomalies in narrative text where unusual word choices often signal factual errors, it struggles with mathematical content where correctness depends on logical relationships rather than lexical patterns. The method’s reliance on surface-level similarity makes it poorly suited for detecting the subtle logical inconsistencies that characterize mathematical hallucinations.

### Specialized detection module

The specialized detection module demonstrates a consistent pattern across both pre-trained and fine-tuned variants: strong contradiction detection but weak correctness validation, with this asymmetry becoming extreme in mathematical domains.

Pre-trained LLMs show variable performance depending on architecture and prompting strategy. On WikiBio, ZS prompting generally outperforms CoT, with Phi-3 3.8B achieving the best balance (89.27 nonfactual, 56.26 factual). LLMs such as Mistral 7B maintain competitive factual detection (58.60) but struggle with ranking tasks. The transition to AIME Math reveals the method’s core limitation: while nonfactual detection remains robust across LLMs (85%–93%), factual detection collapses uniformly, with even the best performer (Gemma 7B) reaching only 20.38%.

Fine-tuning on MultiNLI substantially improves WikiBio performance, with Mistral 7B and Phi-3 3.8B both exceeding 92% nonfactual and 65% factual detection. However, this supervised approach fails to transfer to mathematical reasoning. On AIME Math, fine-tuned LLMs replicate the same asymmetric pattern: excellent contradiction detection (>90%) paired with poor correctness validation (<22%).

This consistent asymmetry across fine-tuning paradigms suggests that current NLI approaches, when fine-tuned on general-domain datasets such as MultiNLI, are fundamentally limited in their ability to validate mathematical reasoning, regardless of LLMs’ scale or fine-tuning strategy.

### Contextual consistency module

The contextual consistency module achieves the strongest performance among all methods tested, establishing new benchmarks on WikiBio while simultaneously revealing the persistent challenges of mathematical reasoning validation.

On WikiBio, this module delivers state-of-the-art results across all metrics. GPT-4o sets the highest factual detection at 74.95% (CoT) and 74.11% (ZS), substantially outperforming all previous methods, including fine-tuned baselines. Nonfactual detection consistently exceeds 91% across LLMs, with GPT-4o CoT reaching 94.14%. Even open-weight LLMs achieve competitive performance, with LLaMA 3.1 reaching 70.73% factual detection, outperforming SelfCheckGPT’s best configuration with GPT-3.5 (67.09%). Ranking correlations similarly excel, with LLaMA 8B with CoT achieving the highest score of 78.48%.

Despite this clear superiority in biographical text, the mathematical domain exposes fundamental limitations that persist even for the best-performing approach. While nonfactual detection remains robust (91%–96%), factual detection undergoes severe degradation. Most notably, GPT-4o, the clear leader on WikiBio, drops dramatically to 24.26% (ZS) and 30.58% (CoT). In contrast, our framework with GPT-3.5 achieves 57.62% factual detection (CoT), closely aligning with the SelfCheckGPT ZS baseline (56.95%) and substantially higher than other prompt-based variants. Open-weight LLMs cluster between 24% and 38%, maintaining the consistent pattern observed across all methods.

These results establish the contextual consistency module as the most effective approach for general hallucination detection while confirming that even the best-performing methods face severe limitations when applied to mathematical reasoning tasks. The persistent domain gap suggests that the challenge lies not in the sophistication of the detection method but in fundamental aspects of mathematical validation that current approaches cannot address.

### Overall comparison

Table 3 reveals several cross-method trends that highlight the underlying challenges of hallucination detection across domains. First, all methods show a pronounced asymmetry between nonfactual and factual detection on both datasets. Nonfactual detection remains consistently high (85%–96%) regardless of LLM scale or architecture, suggesting that identifying explicit contradictions or distributional anomalies is comparatively easy. In contrast, factual detection is substantially lower and strongly domain dependent, indicating that validating correctness requires deeper semantic or logical reasoning that current approaches struggle to capture.

Second, the domain gap between WikiBio and AIME Math is systematic and affects every method category. Whether frequency-based, NLI-based, or prompt-based, all methods exhibit a sharp degradation in factual detection on AIME Math, often a 40- to 55-point drop. This suggests that the difficulty is intrinsic to mathematical reasoning, where correctness hinges on domain-specific logical relations rather than surface-level lexical cues.

Third, the ranking metric mirrors the same pattern. LLMs that perform well on WikiBio (such as GPT-4o or LLaMA 3.1 in the prompt-based setting) experience dramatic drops in ranking correlation on AIME Math, indicating that even when contradictions are detectable, LLMs fail to organize mathematical solutions by correctness. This further confirms that existing semantic representations are insufficient for structured reasoning tasks.

Finally, the relative performance gap between methods narrows considerably on AIME Math. The advantages of contextual or semantic embeddings largely disappear, and even the best-performing LLMs converge to a similarly low ceiling of 20%–38% factual detection, underscoring the difficulty of detecting hallucinations in mathematical reasoning.

## Discussion

### Experimental analysis

#### Examining the influences of module capacity

**Figure 3:**
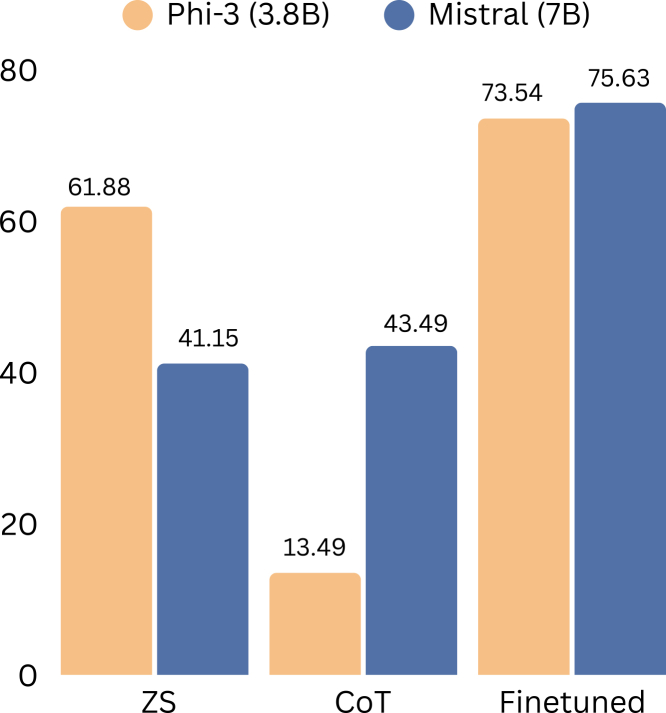
Analysis of LLM capacity impact

Figure 3 shows that increasing LLM capacities, especially in the specialized detection module, significantly improves hallucination detection. Specialized detection modules such as Phi-3 and Mistral outperform their pre-trained counterparts. Table 3 highlights performance variations across module capacities, with the specialized detection module consistently surpassing other methods, achieving top scores for nonfactual, factual, and ranking on both the WikiBio and AIME Math datasets. Among contextual consistency modules, GPT-4o with CoT prompting and GPT-3.5 with ZS prompting show strong performance, emphasizing the importance of LLM size and fine-tuning in hallucination detection (see Note S4).

#### Challenges and anomalies in performance

The specialized detection module analysis reveals some interesting findings. On the AIME Math dataset, RoBERTa-large fails in pre-trained settings due to its limited context window. Also in the AIME Math dataset, significant gaps between nonfactual (AUC-PR), factual (AUC-PR), and ranking (PCC) scores exacerbated the dataset’s imbalance, stemming from the LLMs’ limitations in the complex mathematics domain. Notably, Gemma (7B) in the ZS pre-trained LLM matches Phi-3 (3.8B) fine-tuned scores, most likely due to Gemma’s robust architecture and the dataset’s imbalanced nature, which limits sensitivity to LLM-specific optimizations.

### Analysis for the mathematical domain

The dramatic performance drop observed when transitioning from WikiBio to AIME Math across all SelfCheck-Eval components (results) raises a fundamental question: are these failures inherent to black-box approaches, or can specialized fine-tuning paradigms overcome the mathematical reasoning challenge? We evaluate three fine-tuning strategies targeting different aspects of mathematical reasoning: logical inference (LogiQA 2.0^35^), preference learning (RewardMath^36^), and process supervision (PRM800K^22^ and Math-Shepherd^37^). Following Li et al.,^21^ we frame hallucination detection as a binary classification task and report precision, recall, and F1-score as primary metrics. While Li et al.^21^ focus on a taxonomy of hallucination types, our goal is to examine how different paradigms transfer to mathematical reasoning for the fundamental binary distinction between hallucinated and non-hallucinated outputs. Our analysis disentangles performance across the two classes (hallucinated versus non-hallucinated), providing clearer insights into LLM behavior. For consistency with prior evaluations, we also report AUC scores.

#### NLI-based fine-tuning of LLMs for hallucination detection (LogiQA 2.0)

Mathematical errors are fundamentally logical inconsistencies that can be detected through entailment reasoning. Logical inference represents a natural candidate for transfer learning in hallucination detection: both require distinguishing valid from invalid reasoning. We fine-tune four LLMs on LogiQA 2.0,^35^ a large-scale NLI dataset designed to capture logical patterns beyond single sentences, and test their transfer to mathematical hallucination detection. A detailed description of the datasets can be found in Notes S2 and S3.

The results (Table 4) reveal both the potential and the limitations of NLI-based transfer. All LLMs achieve high AUC values for hallucination classification (79%–93%), with F1-scores ranging from 0.36 (Phi-3) to 0.87 (Qwen2.5-Math). However, performance deteriorates sharply for the non-hallucination class: AUC values fall below 25%, and F1-scores remain below 0.35. This indicates that NLI fine-tuning promotes a conservative detection strategy in which LLMs default to labeling uncertain cases as hallucinated. The behavior varies across architectures. LLaMA3-8B provides the most balanced transfer, with very high precision for hallucination detection (0.94) and the strongest non-hallucination F1-score (0.33). Qwen2.5-Math-7B is highly sensitive to hallucinations (F1 = 0.87, recall = 0.89) but almost unable to validate correct reasoning (F1 = 0.11). Phi-3 takes the opposite stance, showing high non-hallucination recall (0.86) but very low hallucination recall (0.23). While NLI-based fine-tuning equips LLMs with strong contradiction detection, it does not transfer to validating correctness in mathematical reasoning.

**Table 4:** Evaluation results of LLMs on hallucination detection for the logic NLI task

LLM	P(Hallu)	R (Hallu)	F1 (Hallu)	P (non-Hallu)	R (non-Hallu)	F1 (non-Hallu)	AUC (Hallu)	AUC (non-Hallu)
Llama3-8B	**0.94**	0.52	0.67	**0.21**	0.80	**0.33**	**93.26**	22.60
Qwen2.5-7B	0.81	0.59	0.68	0.06	0.15	0.08	79.45	10.22
Qwen2.5-Math-7B	0.86	**0.89**	**0.87**	0.12	0.09	0.11	84.19	12.29
Phi-3	0.91	0.23	0.36	0.15	**0.86**	0.26	90.42	**24.01**

Metrics include precision (P), recall (R), and F1 for both hallucination and non-hallucination classes, as well as AUC scores (in %). The best result is shown in bold.

#### Reward models for hallucination detection

Preference-based fine-tuning can capture the subtle quality differences between valid and invalid mathematical reasoning. Reward modeling has emerged as a key paradigm for aligning LLMs with human expectations, as it leverages preference signals rather than categorical labels.^38^ This makes it particularly relevant for hallucination detection, where errors are not always strictly factual but often involve issues of plausibility, coherence, or logical soundness. The RewardMath framework^36^ extends this principle to mathematical reasoning, where hallucinations typically take the form of invalid steps or unsupported conclusions. In this setting, reward models act as evaluators of reasoning quality, distinguishing between valid and invalid solution paths and enabling a finer-grained form of hallucination detection that goes beyond surface correctness.

The results (Table 5) show that the reward-based approach achieves consistently high AUC values for hallucination detection (86.5%–88.3%). LLaMA3-8B obtains the strongest hallucination classification (F1 = 0.88), followed by Qwen2.5-Math-7B (0.77). In contrast, Mistral-7B (0.50) and Qwen2.5-7B (0.32) perform considerably worse. Performance on the non-hallucination class, however, remains weak: precision ranges from 0.13 to 0.21, recall varies widely (0.18–0.88), and F1 never exceeds 0.25. AUC values for the non-hallucination class also remain low (13.4%–16.6%). Reward-based LLMs are effective at flagging flawed reasoning but struggle to reliably recognize correct solutions, reproducing the same asymmetric pattern observed with NLI-based approaches.

**Table 5:** Evaluation results of fine-tuned LLMs on RewardMATH on hallucination detection

LLM	P (Hallu)	R (Hallu)	F1 (Hallu)	P (non-Hallu)	R (non-Hallu)	F1 (non-Hallu)	AUC (Hallu)	AUC (non-Hallu)
Mistral-7B	0.85	0.36	0.50	0.13	0.61	0.22	86.50	13.39
Qwen2.5-7B	**0.91**	0.19	0.32	0.15	**0.88**	**0.25**	**88.30**	13.92
Qwen2.5-Math-7B	0.86	0.70	0.77	0.14	0.31	0.20	86.47	15.67
Llama3-8B	0.87	**0.89**	**0.88**	**0.21**	0.18	0.19	88.07	**16.58**

Metrics include precision (P), recall (R), and F1 for both hallucination and non-hallucination classes, as well as AUC (%). The best result is shown in bold.

#### PRM for hallucination detection

Step-level supervision can address error propagation in multi-step mathematical reasoning. Reward models assess the overall quality of generated outputs, but they remain limited when reasoning unfolds across multiple steps. In mathematical problem solving, errors often emerge gradually in intermediate steps rather than appearing only in the final answer. PRMs address this limitation by assigning evaluative signals at the level of intermediate reasoning steps, thereby complementing or replacing outcome-based supervision. The idea was introduced by Uesato et al.,^39^ who showed that rewarding intermediate correctness improves reasoning reliability. Subsequent work, most notably Lightman et al.,^22^ formalized this principle by providing a large-scale dataset of human-annotated step-level feedback, namely PRM800K, and demonstrated that process supervision mitigates compounding errors in multi-step reasoning. This approach is especially relevant to our setting, where AIME solutions are naturally decomposed into step-by-step traces. Hallucinations often take the form of subtle errors in intermediate transitions rather than overt mistakes in the final result. PRMs make it possible to estimate the probability that each reasoning step introduces a hallucination and to aggregate these signals into a structured diagnostic of reasoning quality.

To evaluate PRMs for hallucination detection, we fine-tuned LLMs on two complementary datasets: PRM800K,^22^ which provides step-level human annotations of candidate solutions, and Math-Shepherd,^37^ which automatically generates process-level labels for large-scale supervision. Both datasets are designed to capture intermediate reasoning quality rather than only final answers, making them well suited to process-level hallucination detection. The results in Table 6 show that PRM fine-tuning yields strong performance in hallucination detection, with F1 (Hallu) approaching or exceeding 0.90 in several settings. LLaMA3-8B on PRM800K reaches 0.88, Qwen2.5-Math-7B on PRM800K attains 0.93, and LLaMA3-8B on Math-Shepherd achieves 0.93. High recall for the hallucination class, often close to 1.0 as in Qwen2.5-Math-7B on PRM800K and LLaMA3-8B on Math-Shepherd, confirms that PRMs are highly effective at flagging flawed reasoning steps.

**Table 6:** Evaluation results of PRM fine-tuned LLMs (Math-Shepherd and PRM800k) for hallucination detection

LLM	P (Hallu)	R (Hallu)	F1 (Hallu)	P (non-Hallu)	R (non-Hallu)	F1 (non-Hallu)	AUC (Hallu)	AUC (non-Hallu)
Llama3-8B (PRM800k)	0.85	0.92	0.88	0.01	0.07	0.01	79.25	9.44
Qwen2.5-Math-7B (PRM800k)	0.86	**1.00**	**0.93**	0.00	0.00	0.00	81.13	9.94
Qwen2.5-7B (PRM800k)	0.85	0.88	0.87	0.03	0.02	0.02	**83.36**	10.75
Qwen2.5-Math-7B (Math-Shepherd)	0.76	0.14	0.24	0.12	**0.72**	0.20	82.52	**13.89**
Qwen2.5-7B (Math-Shepherd)	0.83	0.20	0.33	0.13	0.74	**0.22**	82.89	10.25
Llama3-8B (Math-Shepherd)	**0.86**	1.00	0.93	**0.50**	0.01	0.01	81.36	10.61

The best results for each metric are highlighted in bold. Metrics include precision (P), recall (R), and F1 for both hallucination and non-hallucination classes, as well as AUC (%).

However, this strength comes at the expense of non-hallucination detection. Across all settings, F1(non-Hallu) remains very low, never exceeding 0.22, with the best result obtained by Qwen2.5-7B on Math-Shepherd. Precision for the non-hallucination class is nearly zero in several configurations, for instance, LLaMA3-8B on PRM800K with 0.01. This imbalance suggests that PRMs adopt an overly conservative strategy, systematically favoring hallucination classification and producing frequent false positives when evaluating valid reasoning.

Process-level supervision, therefore, enables near-perfect sensitivity to hallucinated reasoning but struggles to develop complementary capabilities for validating correct solutions, representing the most extreme manifestation of the conservative bias observed across all approaches.

#### The mathematical domain challenge

Across all three specialized paradigms, NLI fine-tuning, reward modeling, and process supervision, we observe a consistent pattern of asymmetric performance: while LLMs achieve high or even near-perfect sensitivity to hallucinated content (F1 for hallucination detection is often >0.9), they consistently struggle with validating correct reasoning (F1 for non-hallucination detection rarely exceeds 0.25, with most results <0.20). This mirrors the behavior observed in SelfCheck-Eval, suggesting that the mathematical domain poses fundamental challenges that require better training methodology or architectural choice.

When we compare these specialized approaches to our original module on AIME Math, the pattern is remarkably consistent. The contextual consistency module achieved 30.58% factual detection with GPT-4o, while our best specialized approaches reached similar levels (such as Qwen2.5-Math-7B on RewardMath with F1 = 0.20 for non-hallucinations). This convergence across fundamentally different approaches suggests the limitation is domain inherent rather than method specific.

Interestingly, LLMs specialized in mathematical reasoning do not perform better on the non-hallucination class under process supervision. Qwen2.5-Math-7B, for example, achieves near-perfect recall for hallucinations (1.0 on PRM800K) but entirely fails to recognize correct reasoning steps (F1(non-Hallu) = 0.00). By contrast, its non-specialized counterpart Qwen2.5-7B, despite weaker hallucination performance, achieves a higher balance with F1(non-Hallu) = 0.22 on Math-Shepherd. This suggests that mathematical specialization amplifies the conservative bias of PRMs: rather than learning to validate correct reasoning, specialized LLMs default to classifying nearly all cases as hallucinations.

The failure appears rooted in the fundamental difference between fine-tuning domains and mathematical reasoning tasks. LLMs fine-tuned on logical inference datasets such as LogiQA 2.0 learn to identify contradictions in natural language, but these skills do not transfer effectively to mathematical content, where correctness depends on precise logical relationships and multi-step reasoning chains. Reward models trained on mathematical solution preferences can distinguish quality differences but struggle with subtle reasoning errors. PRMs, despite step-level supervision, adopt overly conservative strategies that flag valid reasoning as potentially problematic.

Our findings demonstrate that current approaches face significant limitations when applied to mathematical reasoning, regardless of the fine-tuning paradigm employed. The consistent performance ceiling across NLI fine-tuning, reward modeling, and process supervision suggests that effective mathematical hallucination detection may require fundamentally different methods explicitly designed for formal reasoning domains, potentially integrating symbolic verification, proof-checking, or hybrid neuro-symbolic techniques. The convergence of results across diverse fine-tuning paradigms thus represents a robust finding about the inherent difficulty of applying statistical learning methods to domains that demand rigorous logical validation.

Although this work focuses on detection, our results suggest practical directions for mitigating mathematical hallucinations. We observe that many errors originate in intermediate reasoning steps rather than only in final answers, indicating that step-level verification of calculations and transformations could prevent error propagation in multi-step solutions. Furthermore, our contextual consistency experiments show that comparing multiple LLM-generated solutions reliably exposes hallucinations. This suggests a mitigation strategy in which several candidate solutions are generated and the final output is selected based on cross-solution agreement, thereby filtering out unreliable responses before presenting them to users. Overall, the mechanisms effective for detecting hallucinations, step-level verification, and cross-solution consistency can also serve as practical strategies for reducing hallucinated mathematical outputs.

### Conclusions

We introduce SelfCheck-Eval, a systematic framework for evaluating hallucination detection across domains, and the AIME Math Hallucination benchmark for mathematical reasoning evaluation. Our analysis reveals fundamental domain-dependent limitations in current approaches. Our evaluation demonstrates that mathematical reasoning poses unique challenges for hallucination detection. While all methods achieve robust performance on biographical content, they exhibit consistent asymmetric behavior in mathematical domains. In fact, all methods are able to detect incorrect solutions while systematically failing to validate correct reasoning. This pattern persists across NLI fine-tuning, preference learning, and process supervision, affecting even mathematics-specialized LLMs. The consistent performance convergence across diverse fine-tuning paradigms suggests that mathematical correctness requires rigorous logical validation that transcends current statistical learning approaches. Unlike biographical facts validated through distributional patterns, mathematical reasoning demands domain-specific verification mechanisms. Our detection findings suggest two practical mitigation directions: verifying individual reasoning steps to catch errors early and using consensus across multiple generated solutions to identify reliable outputs.

### Limitations

Our evaluation focuses on competition-level mathematical reasoning through AIME problems versus biographical content from WikiBio. While this comparison reveals fundamental differences between domains, the generalizability to other formal reasoning contexts such as physics, computer science, and symbolic logic requires further investigation. The AIME problems represent competition-level mathematics that require creative problem-solving strategies, which may not reflect all mathematical reasoning scenarios encountered in practical applications. Additionally, our binary classification approach (hallucinated versus non-hallucinated) may not capture the nuanced spectrum of mathematical errors, from minor computational mistakes to fundamental conceptual misunderstandings. Future work could incorporate additional evaluation metrics beyond AUC-PR and PCC to enable a more comprehensive assessment of performance. Our findings suggest several critical research directions: developing hybrid approaches that integrate statistical detection with symbolic verification for mathematical domains, extending evaluation to broader formal reasoning contexts, and investigating domain-adaptive architectures specifically designed for rigorous logical validation.

## Resource availability

### Lead contact

Requests for further information and resources should be directed to and will be fulfilled by the lead contact, Gollam Rabby (gollam.rabby@l3s.de).

### Materials availability

This study did not generate new unique reagents or materials.

### Data and code availability

The open-source dataset used for this study is accessible through Hugging Face. Interested readers and researchers can obtain the dataset by visiting the following links: Hugging Face: https://huggingface.co/datasets/tourist800/AIME_Hallucination_Detection and Hugging Face: https://huggingface.co/datasets/potsawee/wiki_bio_gpt3_hallucination. The study was carried out exclusively with open-source software packages. All scripts, outcomes, post-processed datasets, and features will be accessible to the public at Zenodo: https://doi.org/10.5281/zenodo.19431203.^40^

## Acknowledgments

We acknowledge the support of the KISSKI project (funding no. 01IS22093C) for providing computational resources, which will enable us to extend this research in the future. G.G.T. acknowledges FOSSR (Fostering Open Science in Social Science Research), funded by the European Union-NextGenerationEU under NRRP grant agreement no. MUR IR0000008.

## Author contributions

This work was carried out through close collaboration among all authors. G.R. designed the experiment, led the experiment, and wrote the manuscript. D.M. and G.G.T. were responsible for implementing and conducting the experiments and writing the manuscript. S.A. and S.V. played a significant role in conceiving the experiment design and contributed to the writing of the manuscript.

## Declaration of interests

The authors declare no competing interests.

## Declaration of generative AI and AI-assisted technologies in the writing process

During the preparation of this work, the authors used ChatGPT to improve grammatical clarity and correct language errors. After using this tool, the authors reviewed and edited the content as needed and take full responsibility for the content of the publication.

## Supplemental information

Document S1. Tables S1–S5 and Notes S1–S5

Document S2. Article plus supplemental information
