# Supplementary material for: SelfCheck-Eval: A multi-module framework for zero-resource hallucination detection in large language models
Source: Patterns (N Y). 2026 Jun 5;7(6):101569. doi: 10.1016/j.patter.2026.101569 (PMC13280721; doi:10.1016/j.patter.2026.101569)
Supplement: Document S1. Tables S1–S5 and Notes S1–S5 [file mmc1.pdf]

Patterns, Volume *7*

Supplemental information

SelfCheck-Eval: A multi-module framework for zero-resource hallucination detection in large language models

Diyana Muhammed, Giusy Giulia Tuccari, Gollam Rabby, Sören Auer, and Sahar Vahdati

Note S1: Prompts in Experiment

Complete zero-shot and chain-of-thought prompt templates used by the Specialized Detection and Contextual Consistency modules.

Specialized Detection module on Pretrained LLMs

Zero-shot prompt

Statement 1: `{sentence}`

Statement 2: `{sample}`

Task: Analyze if these statements contradict or agree.

Instructions: Answer with `contradict` or `agree`.

Chain-of-thought prompt

Statement 1: `{sentence}`

Statement 2: `{sample}`

Task: Let's reason step by step to see if these statements are related.

Instructions: Consider whether the second statement logically follows from or contradicts the first one. Answer with `contradict` or `entailment`.

Contextual Consistency module

Zero-shot prompt

Context: `{context}`

Sentence: `{sentence}`

Task: Is the sentence supported by the context above?

Instructions: Answer `Yes` or `No`.

Answer:

Chain-of-thought prompt

Context: `{context}`

Sentence: `{sentence}`

Task: Check if the sentence is supported by the context.

Instructions: Think step by step: 1. Understand what the sentence claims. 2. Check if the context provides evidence for this claim. 3. Decide if the sentence is supported by the context.
After reasoning, respond with ONLY one word: `Yes` or `No`.

Answer:

Note S2: Hallucination Detection Datasets

Description of the AIME and WikiBio hallucination detection datasets, including column structure and annotation schemes (see Tables S1 and S2).

AIME Hallucination Detection Dataset

This dataset is designed for detecting hallucinations in LLMs, particularly for complex mathematical problems. It can be used for evaluation and related research. The dataset contains the LLM's initial response and sampled response, along with their corresponding labels. Column headers and a description of the dataset are provided in Table S1.

Column Name	Description
Year	Year in which the problem appeared in AIME.
Set	The set in which the problem appeared.
Problem Number	Problem number.
URL	URL for the problem.
Problem Statement	The mathematical problem/query.
Exact Answer	The exact final answer to the problem.
Solution 1-13	Solutions provided by human experts.
LLM Solution (gpt-4o)	LLM's generated solution.
Exact Answer (gpt-4o)	Exact answer of LLM's output.
Annotation	0: Accurate<br>1: Minor_inaccurate<br>2: Major_inaccurate
Sampled Responses	LLM's sampled outputs.

Table S1. AIME columns and descriptions

WikiBio Hallucination Detection Dataset

This dataset includes Wikipedia-like passages generated by GPT-3 (text-davinci-003) with human annotations for sentence-level accuracy. It is used to evaluate hallucination detection in LLMs. Column headers and descriptions are provided in Table S2.

Column Name	Description
gpt3_text	GPT-3 generated passage.
wiki_bio_text	Actual Wikipedia passage (first paragraph)
gpt3_sentences	gpt3_text split into sentences using spacy
wiki_bio_test_idx	ID of the concept/individual from the original wikibio dataset (testset)
Annotation	human annotation at the sentence level<br>Accurate<br>Minor_inaccurate<br>Major_inaccurate
gpt3_text_samples	list of 20 sampled passages

Table S2. WikiBio columns and descriptions.

Note S3: Additional Datasets

Description of the four specialized fine-tuning datasets LogiQA 2.0, RewardMATH, Math-Shepherd, and PRM800K with example instances and training hyperparameters.

Dataset LogicQA

The NLI version of the LogiQA 2.0 dataset consists of 39k premise–hypothesis pairs annotated as *Entailed* or *Not Entailed.* Unlike standard NLI datasets, LogiQA 2.0 emphasizes logical inference beyond the sentence level, making it suitable for evaluating transfer to multi-step reasoning tasks in AIME-MATH. An example instance from LogiQA2NLI is shown in Listing 1.

Listing 1: Example instance from the LogiQA2NLI dataset.

```
{
  "label": "not entailed",
  "major_premise": [
    "A, B, and C have three balls, one is red, one is blue, and
       the other is yellow"
  ],
  "conclusion": "A is red, B is blue, C is yellow",
  "minor_premise": "C is bigger than the yellow ball, A and the
     blue ball are not the same size, and the blue ball is
     smaller than C"
}
```

In our experiments, we fine-tuned several LLMs on the QA2NLI from LogiQA 2.0. The selected LLMs include:

- **LLaMA3-8B**, as a general-purpose baseline;
- **Qwen2.5-7B**, chosen for its instruction-following and long-context reasoning capabilities;
- **Qwen2.5-Math-7B**, with prior fine-tuning on mathematical chain-of-thought supervision;

- **Phi-3**, based on its strong performance in earlier detection experiments.

To ensure comparability, all LLMs were fine-tuned under identical hyperparameter settings: 6 epochs, a learning rate of 1×10^{-5}, and the Adam optimizer. This configuration was found to be the most effective in our experimental setup.

Dataset RewardMATH

The RewardMATH dataset consists of 4,830 examples derived from 483 problems. Each problem is paired with one correct and multiple incorrect solutions, spanning seven domains: Algebra, Intermediate Algebra, Prealgebra, Number Theory, Precalculus, Geometry, and Counting & Probability. The following excerpts (Listing 2 and 3) illustrate the structure of RewardMATH examples (simplified). We report both a `chosen` (preferred) and a `rejected` (non-preferred) solution.

Listing 2: RewardMATH example with a chosen solution.

```
{
  "problem": "$441+2(21)(19)+361=x$. Solve for $x$.",
  "eval_solution": [
    "Simplify: 2(21)(19) = 798.",
    "Equation becomes 441 + 798 + 361 = x.",
    "This equals (21+19)^2 = 1600.",
    "Therefore, x = 1600."
  ],
  "solution_type": "chosen",
  "level": "Algebra"
}
```

Listing 3: RewardMATH example with a rejected solution.

```
{
  "problem": "$441+2(21)(19)+361=x$. Solve for $x$.",
  "eval_solution": [
    "Calculate 2(21)(19) = 800.",
    "Equation becomes 441 + 800 + 361 = 1602.",
    "Therefore, x = 1602."
  ],
  "solution_type": "rejected",
  "level": "Algebra"
}
```

All reward models were trained using the Hugging Face TRL RewardTrainer (https://huggingface.co/docs/trl/main/en/reward_trainer), for three epochs with a learning rate of 1×10^{-5}.

Dataset MathShepherd

The MathShepherd dataset[?] provides large-scale process-level supervision by automatically generating step-level labels. Each intermediate step in a reasoning trace is scored based on whether it can lead to the correct solution. The dataset contains 445k instances (422k train and 22.2k test), enabling scalable process supervision without requiring manual annotation. An example from MathShepherd is reported in Listing 4.

Listing 4: Example instance from the MathShepherd dataset.

```
{
  "problem": "If two distinct numbers are randomly chosen from
     the set {1, 2, 3, 4, 5},
  what is the probability that the smaller number is a divisor of
      the larger number?
  Express your answer as a common fraction.",
  "steps": [
    "Step 1: There are a total of C(5,2)=10 pairs of numbers that
        can be chosen.",
    "Step 2: List them all: (1,2), (1,3), (1,4), (1,5), (2,3),
       (2,4), (2,5), (3,4), (3,5), (4,5).",
    "Step 3: Valid pairs are (1,2), (1,3), (1,4), (1,5), (2,4).",
    "Step 4: So, there are 5 pairs that satisfy the condition.",
    "Step 5: Probability = 5/10 = 1/2."
  ],
  "labels": [true, true, true, true, true]
}
```

LLMs were fine-tuned on 30% of the official training split, as preliminary experiments showed that using the full dataset provided no additional improvement. We fine-tuned for three epochs, with a learning rate of 1×10^{-5}, the Adam optimizer, and a fixed random seed of 10, using the Hugging Face TRL `PRMTrainer` (https://huggingface.co/docs/trl/main/prm_trainer).

Dataset PRM800K

The PRM800K dataset provides step-level supervision: each intermediate reasoning step is annotated as `true` (correct) or `false` (incorrect). An example instance is shown in Listing 5.

Listing 5: Example instance from the PRM800K dataset.

```
{
  "prompt": "How many seconds are in 7.8 minutes?",
  "completions": [
    "7.8 minutes is the same as 7 minutes and 0.8 minutes.",
    "Right, and since there are 60 seconds in a minute, then
       there are 60 * 7 = 420 seconds in 7 minutes.",
    "And since there are 60 seconds in a minute, then there are
       60 * 0.8 = 48 seconds in 0.8 minutes.",
    "So, in total, there are 420 + 48 = 468 seconds in 7.8
       minutes.",
    "Right. Let's check our work. 7.8 minutes is the same as 7
       minutes and 0.8 minutes."
```

```
  ],
  "labels": [true, true, true, true, false]
}
```

All LLMs were fine-tuned using the Hugging Face TRL `PRMTrainer` (https://huggingface.co/docs/trl/main/prm_trainer) for three epochs, with a learning rate of 1×10^{-5}, the Adam optimizer, and a fixed random seed of 10.

Note S4: Real-World Application Case Study

Preliminary evaluation of SelfCheck-Eval on ChatGPT-generated responses to general-knowledge queries (see Table S3).

**Query**	**LLM Response**	**Sampled Responses**	**Semantic N-gram**	**NLI Method**	**Prompt Method**
Who was Nikola Tesla, and what were his major contributions to science?	Nikola Tesla was a Serbian-American inventor...	Tesla advanced AC ...; Tesla invented ...; Tesla's later life ...	**0.33**	**0.11**	**0.09**
Who was the first person to propose the theory of evolution, and what was their main idea?	The first person to propose the theory of evolution was Charles Darwin...	Charles Darwin introduced ...; Lamarck proposed ...; Mendel's work focused ...	**0.71**	**0.89**	**0.93**

Method Descriptions:

Semantic N-gram: Evaluates responses using N-gram probabilities to compute hallucination scores and assess factuality.
NLI Method: Sampled passages S_n merge with response r_i, generating logits for entailment, contradiction, or neutral scores.
Prompt Method: CoT prompting checks context support, classifying sentences as "yes" or "no" and computes hallucination scores.

Table S3. Real-world hallucination detection on ChatGPT responses. Lower scores indicate higher confidence in factual accuracy.

To assess SelfCheck-Eval's behavior beyond controlled benchmarks, we conducted a preliminary evaluation on ChatGPT-generated responses for general knowledge queries. While limited in scope, this analysis provides initial insights into the framework's applicability to uncontrolled scenarios.

Table S3 presents two illustrative cases with contrasting characteristics. The Tesla query represents a straightforward factual case where all three methods converge on low hallucination scores (0.09-0.33), indicating consistent content across responses. The evolution query demonstrates a different pattern: the main response attributes the proposal of evolutionary theory to Charles Darwin, while the sampled responses mention other figures such as Lamarck and Mendel. This divergence in content leads all methods to assign higher scores (0.71-0.93), indicating detected inconsistencies. These results demonstrate that the methods can provide consistent assessments across different types

of content and respond to varying degrees of consensus among sampled responses. The convergence of all three methods in both cases suggests that the framework maintains its detection capabilities when applied to realistic scenarios outside controlled benchmarks. This preliminary exploration indicates promising directions for real-world deployment, while highlighting the need for more extensive validation across diverse query types and domains.

Note S5: Experimental Results

Detailed results of the Specialized Detection module across LLMs, sizes, and prompting strategies on the WikiBio and AIME datasets (see Tables S4 and S5).

Results on WikiBio Dataset

Method	LLM	Size	Prompt	NonFact	Factual	Ranking
Pre-trained	T5 small	60M	ZS	70.04	25.55	-9.78
Pre-trained	GPT2	124M	ZS	71.67	29.26	0.68
Pre-trained	Roberta Large	355M	ZS	76.44	31.20	6.07
Pre-trained	Phi-3	3.8B	ZS	89.27	56.26	61.88
Pre-trained	Llama 3.1	8B	ZS	78.90	37.12	34.25
Pre-trained	Mistral	7B	ZS	86.13	58.60	41.15
Pre-trained	Gemma	7B	ZS	75.41	30.87	4.80
Pre-trained	T5 small	60M	CoT	69.97	24.79	-10.95
Pre-trained	GPT2	124M	CoT	72.01	30.59	3.55
Pre-trained	Roberta Large	355M	CoT	73.86	31.47	13.02
Pre-trained	Phi-3	3.8B	CoT	75.08	29.67	13.79
Pre-trained	Llama 3.1	8B	CoT	63.88	21.75	-17.11
Pre-trained	Mistral	7B	CoT	81.91	46.10	43.49
Pre-trained	Gemma	7B	CoT	70.97	25.99	-7.62
Fine-tuned	Phi-3	3.8B	-	92.87	65.25	73.54
Fine-tuned	Llama 3.1	8B	-	76.85	29.71	8.91
Fine-tuned	Mistral	7B	-	92.68	67.10	75.63
Fine-tuned	Gemma	7B	-	83.47	43.12	50.98

Table S4. Results for Pre-trained and Fine-tuned Methods on WikiBio Dataset.

Results on AIME Dataset

Method	LLM	Size	Prompt	NonFact	Factual	Ranking
Pre-trained	GPT2	124M	ZS	85.92	13.19	-2.22
Pre-trained	Phi-3	3.8B	ZS	90.34	19.16	12.75
Pre-trained	Llama 3.1	8B	ZS	87.76	21.10	3.16
Pre-trained	Mistral	7B	ZS	91.33	17.11	2.57
Pre-trained	Gemma	7B	ZS	93.38	20.38	21.76
Pre-trained	GPT2	124M	CoT	86.58	14.25	1.65
Pre-trained	Phi-3	3.8B	CoT	87.76	21.10	3.16
Pre-trained	Llama 3.1	8B	CoT	85.33	13.13	-4.07
Pre-trained	Mistral	7B	CoT	91.33	17.11	2.57
Pre-trained	Gemma	7B	CoT	90.07	13.88	0.5
Fine-tuned	Phi-3	3.8B	-	93.38	20.38	21.76
Fine-tuned	Llama 3.1	8B	-	79.63	9.74	-15.44
Fine-tuned	Mistral	7B	-	92.91	17.37	20.05
Fine-tuned	Gemma	7B	-	82.58	10.70	-16.77

Table S5. Results for Pre-trained and Fine-tuned Methods on AIME Dataset.
